# Effects of stochastic vestibular stimulation on cognitive performance in children with ADHD

**DOI:** 10.1007/s00221-023-06713-7

**Published:** 2023-10-09

**Authors:** Erica Jostrup, Marcus Nyström, Emma Claesdotter-Knutsson, Pia Tallberg, Peik Gustafsson, Oskar Paulander, Göran Söderlund

**Affiliations:** 1 Child and Adolescent Psychiatry, Department of Clinical Sciences, Lund University, Lund, Sweden; 2 Lund University Humanities Lab, Lund, Sweden; 3 Outpatient Department, Child and Adolescent Psychiatry Clinic, Region Skåne, Lund, Sweden; 4 Gullbrandstorp Ingenjörsbyrå, Halmstad, Sweden; 5 Faculty of Teacher Education Arts and Sports, Western Norway University of Applied Sciences, Sogndal, Norway; 6 Department of Education and Special Education, University of Gothenburg, Gothenburg, Sweden

**Keywords:** White noise, Stochastic vestibular stimulation, Working memory, Cognitive performance, Reaction time variability, Attention-deficit hyperactivity disorder

## Abstract

Previous work has shown that exposure to auditory white noise (WN) can improve cognitive performance in children with ADHD, but it is unknown whether this improvement generalizes to other sensory modalities. To address this knowledge gap, we tested the effect of Stochastic Vestibular Stimulation (SVS) on cognitive performance and reaction time (RT) variability in two groups: children with ADHD and typically developing children (TDC). Children with ADHD (N=42) and TDC (N=28) performed three cognitive tasks (Spanboard, Word Recall and N-back tasks) at two different occasions, with and without exposure to SVS, in a double blinded design. The results showed no main effects of SVS on neither performance nor RT variability for children in any of the groups, and no interactions between SVS and group. Based on these results we conclude that, using our stimulation protocol, the positive effects of WN exposure on cognition in children with ADHD do not generalize to Stochastic Vestibular Stimulation.

**Supplementary Information:**

The online version contains supplementary material available at 10.1007/s00221-023-06713-7.

## Introduction

There is compelling evidence that exposure to auditory white noise can lead to improved cognitive performance in various cognitive tasks (Pickens et al. 2019). White noise is described as a continuous, smooth, and random signal that carries no meaningful information to the participant or the task at hand and can be applied in any modality (Sikström and Söderlund 2007). More generally, noise can be described as unwanted or random variance that typically degrades the quality of a signal, and is often described by its statistical properties, e.g., its distribution over the power spectrum of frequencies or in ‘colors’, e.g., white, pink or brown noise (Oppenheim 1999).

Auditory noise exposure has been studied in both neurotypical people (Awada et al. 2022) and in relation to neurodevelopmental disorders, where particularly young people with attention deficits and/or attention-deficit hyperactivity disorder (ADHD) have shown noise benefit in cognitive performance (Baijot et al. 2016; Lin 2022; Söderlund et al. 2016). ADHD is the most common neurodevelopmental disorder, affecting up to 5% of children and 2.5% of adults (Faraone et al. 2021; Willcutt 2012), and is characterized by developmentally inappropriate and impairing inattention, hyperactivity, and impulsivity (American Psychiatric Association 2013). ADHD is characterized by executive function deficits, including working memory, sustained attention, planning, and inhibitory control, leading to impulsive behavior and slower information processing compared to peers (Chamorro et al. 2022). In particular reaction time (RT) variability have been shown to have the largest effect in comparison to healthy controls (Pievsky and McGrath 2018).

When exposed to auditory white noise, children rated as sub-attentive by their teachers have shown better performance on working memory tasks and Go/No-Go tasks (Helps et al. 2014), and children with ADHD show better memory recall capacities when exposed to auditory white noise (Söderlund et al. 2007). The effect of auditory white noise exposure has also been evaluated in comparison with stimulant medication in ADHD where noise exposure outperformed medication in two memory tasks (Söderlund et al. 2016).

The Moderate Brain Arousal (MBA) model by Sikström and Söderlund (2007) explains why certain types of noise improves cognitive performance in individuals with attention deficits. While the etiology of ADHD is complex, some suggest reduced dopamine (DA) and/or norepinephrine (NE) levels lead to ADHD symptoms (Sharma and Couture 2014). In line with this, the model postulates that brains with excessive levels of internal neural noise due to low dopamine levels, counterintuitively, benefit from external noise exposure that actually increases the signal-to-noise level making the brain work at its maximum (Söderlund and Sikström, 2012). The MBA model suggest that the mechanism behind the noise benefit is due to the phenomenon of stochastic resonance (SR), where stimuli presented under a detection threshold can be detected in the presence of white noise. The noise interacts with the target signal and reinforces weak signals, pushing them over the detection threshold, and thus increases the signal-to-noise ratio (Moss et al. 2004).

The MBA model relies on the assumption that a certain amount of white noise, an individually based moderate noise level, is necessary to yield an optimal signal transmission in the brain, whereas too much or too little noise is disruptive, following an inverted U-shaped curve (Sikström and Söderlund 2007). Taken together, the MBA model predicts that patients with ADHD can be exposed to, and benefit from, noise in several modalities (auditory, visual, tactile). Similarly, the MBA model also suggests that exposing healthy controls to noise will decrease cognitive performance, since noise is added when an optimal brain arousal level is already established, bringing them out in the far (right) side of the inverted U-curve.

While previous research has focused primarily on effects on cognitive performance of auditory noise, it is not clear whether the effects generalize to all sensory modalities. Here we investigate if stochastic electric noise exposure on the vestibular system has a similar effect on cognition. Stochastic Vestibular Stimulation (SVS) have been investigated in various patient groups earlier, such as Parkinson patients and elderly (Herrera-Murillo et al. 2022; Samoudi et al. 2015). SVS is based on an alternating, stochastic, current and is delivered through electrodes placed bilaterally on the mastoid bone, behind the ears, coupled with an electrical generator. The sensory noise stimulation flows via the bones to the vestibular afferent nerve and activates the vestibular system (Utz et al. 2010).

The vestibular system is known for its involvement in maintaining balance and gaze stability (Bigelow and Agrawal 2015) and applying SVS has proven to be effective in assisting balance and posture (Mulavara et al. 2011; Mulavara et al. 2015; Samoudi et al. 2015). Later research suggests that the vestibular system is involved in cognitive processes as well (Hitier et al. 2014), and due to the connectivity of the vestibular system, Ferrè and Haggard (2020) suggest that vestibular stimulation could have a modulatory influence on several neurocognitive functions such as memory, emotional control and visuo-spatial attention. Further example of this is Hilliard et al. (2019), who showed that spatial learning and memory can be enhanced by SVS in healthy adults. The level of spatial working memory capacity moderated the effects, with those having a lower capacity had larger effects of the stimulation. Similar effects of improvement by SVS were found by Voros et al. (2021), who investigated visual thresholds.

**Fig. 1:**
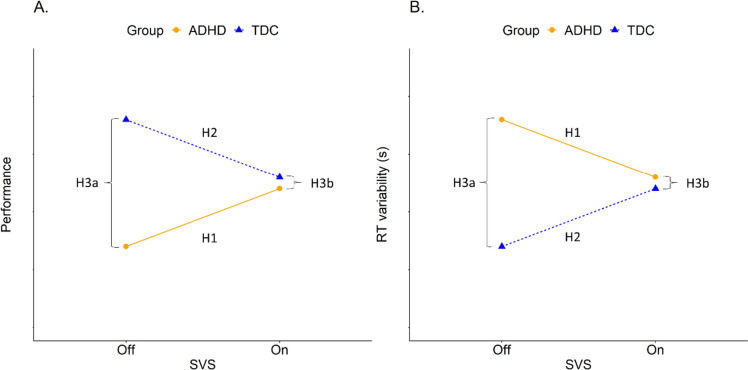
Hypotheses: H1: ADHD children will improve performance **A** and decrease RT variability **B** with SVS, H2: TDC will get impaired performance **A** and increased RT variability **B** by SVS, H3a: TDC has higher performance **A** and lower RT variability **B** than ADHD without SVS, and H3b: the difference between TDC and ADHD will be diminished by SVS (**A** + **B**)

The main aim of the current study was to test the effects of SVS on cognitive performance in children with ADHD and a group of typically developing children (TDC) as comparison. Based on the MBA model we hypothesized that SVS would improve performance and reduce RT variability in children with ADHD (H1) and impair performance and increase RT variability in the TDC group (H2). Based on previous research, we also expected that the TDC group would have higher performance and lower RT variability without SVS (H3a), and that SVS would remove such differences (H3b). These hypotheses are visualized in Fig. 1 below.

## Methods

### Design

To evaluate the effects of vestibular noise (SVS) exposure on cognitive performance three tasks targeting different aspects of cognition were used in a 3 × 2 repeated measures design: a visuo-spatial working memory task (Spanboard), a verbal episodic memory task (Word recall) and a visual N-back task. The tasks were performed on two different occasions, with and without SVS active.

In our original design, we aimed to replicate previous findings of auditory noise on cognitive performance as well as investigate the effects of adding noise in two modalities concurrently, and therefore added auditory noise to half of the Word Recall tests. Unfortunately, due to technical problems, the auditory noise was considered not reliable and is therefore not included in the analysis. We identified a glitch in the headphones leading to irregular, and below calibrated, levels of speech. Therefore, auditory noise exposure will not be described in the method and was excluded from the results.

### Participants

The study included 71 children aged between 8–17 years. Forty-three participants with an ADHD diagnosis (28 males and 15 females, mean age 12.4 years, SD = 2.07) and twenty-eight TDC (12 males and 16 females, mean age 11.2 years, SD = 0.39). Our power calculations for number of participants in the study were based on prior research on auditory noise stimulation, thus indicating the need of at least 26 participants in each group. The TDC participants were recruited from local schools in the district of Skåne. The ADHD group was recruited from the child and adolescent psychiatry clinic in Lund, Sweden, or from parents actively signing up to be part of the study through online advertising.

All participants with ADHD were diagnosed according to the Diagnostic and Statistical Manual of Mental Disorder, 5th Edition [DSM-5] (American Psychiatric Association 2013), according to international guidelines. A child psychiatrist (one of the authors) confirmed the ADHD diagnoses. Furthermore, all participants were screened for attention ability and hyperactive symptoms using the SNAP rating scale (Swanson et al. 2012). The ADHD participants were rated by their parents while the TDC group were rated by their teachers. The SNAP IV rating scores (Table 1) were used to validate the ADHD and the control groups. Participants with an ADHD diagnosis that were on medication all had a minimum of 24 h washout prior to the study.

**Table 1:** Participants’ characteristics and SNAP IV rating on hyperactivity and inattention

	ADHD	TDC	ADHD vs. TDC
Boy/Girl	28/15	12/16	*X*^*2*^(1, *N* = 70)= 5.42, *p* = .020*
Age	12.4 (2.1)	11.2 (0.4)	*t*(91) = − 5.18, *p* < .001***
Hyperactivity	14.9 (6,8)^a^	0.3 (0.9)^b^	*t*(85) = − 19.18, *p* < .001***
Inattention	17.6 (4.8)^a^	1.9 (2.7)^b^	*t*(132) = − 24.34, *p* < .001***
Total (H+I)	32.5 (10.3)^a^	2.2 (3.2)^b^	*t*(102) = − 24.84, *p* < .001***

Mean (standard deviation)

Age: years, *H* hyperactivity, *I* inattention

^a^Parent rated scores

^b^Teacher rated scores

*p ≤ 0.05, ***p ≤ 0.001

### Ethics

Written consent was obtained from the legal guardians of all participants, as well as all from all participants aged 15 years or above. Oral approval from participating children was given before taking part in the study. This study was approved by the Regional Ethics Committee at Lund University (EPN 2021-04444) and registered at clinicaltrials.org under ID NCT03425669. All participants were debriefed after participating in the study and no aversive experiences were reported.

### Test battery

#### Spanboard

In a visuo-spatial working memory task, Spanboard (Söderlund et al. 2016; Westerberg et al. 2004), consisting of a 4 × 4 grid with 16 squares, the participants were asked to remember the location and order of a series of red dots appearing in the grid, one by one, and recall the sequence using the computer mouse. The goal was to reach the highest possible level, i.e., to remember as many dots in a sequence as possible. No feedback of correct or false answers were given.

Every dot was shown for 2250 ms followed by a pause for 750 ms before the next dot appeared making an interstimulus interval (ISI) of 3 s. The experimental assessment started with two dots to be remembered and on every second successive trial another dot was added until the participant made an error on two successive trials on the same level. The reaction times were measured as the time between two mouse clicks. The task took about 5 min to complete.

Dependent variables for this task were: (1) The total number of correctly recalled dots and (2) the standard deviation of reaction time values, henceforth RT variability.

#### Word recall

In a word recall task participants were instructed to orally recall as many words as possible after listening to pre-recorded words being read aloud in a pair of earphones. The word recall task consisted of four repetitions, or lists, on each occasion. The two with auditory noise were excluded from the analysis due to above referred technical problems. The entire task consisted of eight lists with 12 unrelated Swedish nouns in each. The 96 words were selected from a list containing 157 words, previously used in Flodin et al. (2012), and were matched for; (i) frequency of use in the Swedish language using the Swedish Kelly-list (Volodina 2019; Volodina and Kokkinakis 2012), (ii) word length and (iii) number of syllables. The lists contained a combination of both high frequency words and low frequency words.

A balanced latin-square matrix controlled the presentation order of the lists based on the participant id. The recorded words were approximately 1 s each and the words within the lists were presented in random order with a four second pause between each word, giving an ISI of 5 s. The participants were given both written and oral instructions of the task. During the time the lists were presented a fixation cross was displayed in the center of the screen, on which the participant was asked to focus. After each list the participant was asked to orally recall as many of the words possible, in any order. Answers given in plural instead of singular were also considered correct. The task took about 8 min to complete, including the 2 lists that were excluded from the analysis.

A pair of Sennheiser HDA 300 earphones was used to present the speech signal at 81 dB SPL. The calibration of the speech signal and the equipment used in the study was done according to ISO 389-8 (2004) and IEC 60318-2 (1998) and the calibration was done using a Brüel and Kjaer Impulse Precision Sound Level Meter Type 2209 with a 4134 microphone in a 4153 ear-simulator.

The dependent variable for this task was the number of correctly recalled words.

#### N-back

A visual N-back task consisting of three levels of difficulty (0-, 1- and 2-back) were performed as the last task. The stimuli used in the task were twenty 3-dimensional figures built by cubes with the same size. The task required the participant to remember a figure, the target, n-steps (0, 1 and 2) back in a sequence of figures.

In the 0-back task participants had been instructed to press the left arrow key whenever the (pre-chosen) target was shown and the right arrow key for all other stimuli. The 1-back task requested the participant to press the left arrow key for any stimuli shown twice in a row. In the 2-back task the participants were asked to give a response to whether the third image was the same as the first or not. All following figures were to be responded to as either matching or mismatching with the one shown two steps earlier (2-back) and the participant response rate determined the pace of the task. Exceptions from this were the first target(s) in the 1- and 2-back task (one in the 1-back task, two in the 2-back task), which were shown for 2 s. All figures were followed by a fixation cross, shown for 1 s, before the next figure was presented. The response times were measured as the time from when a new figure appeared to any given answer (pressing right/left arrow).

Participants completed a baseline assessment on each level, all including 10 trials, with a combination of written and assisted oral instructions before the experimental assessment started. All assessments were completed in the order of 0-, 1-, and 2-back, making them increasingly harder.

Each level consisted of 30 trials, where 1/3 were targets and 2/3 non-targets. The sequence of the stimuli was randomized for each trial, level, participant, and occasion. The task took about 10 min to complete.

The dependent variables for this task were (1) RT variability and (2) number of incorrect answers.

### Stochastic vestibular stimulation (SVS) protocol

**Fig. 2:**
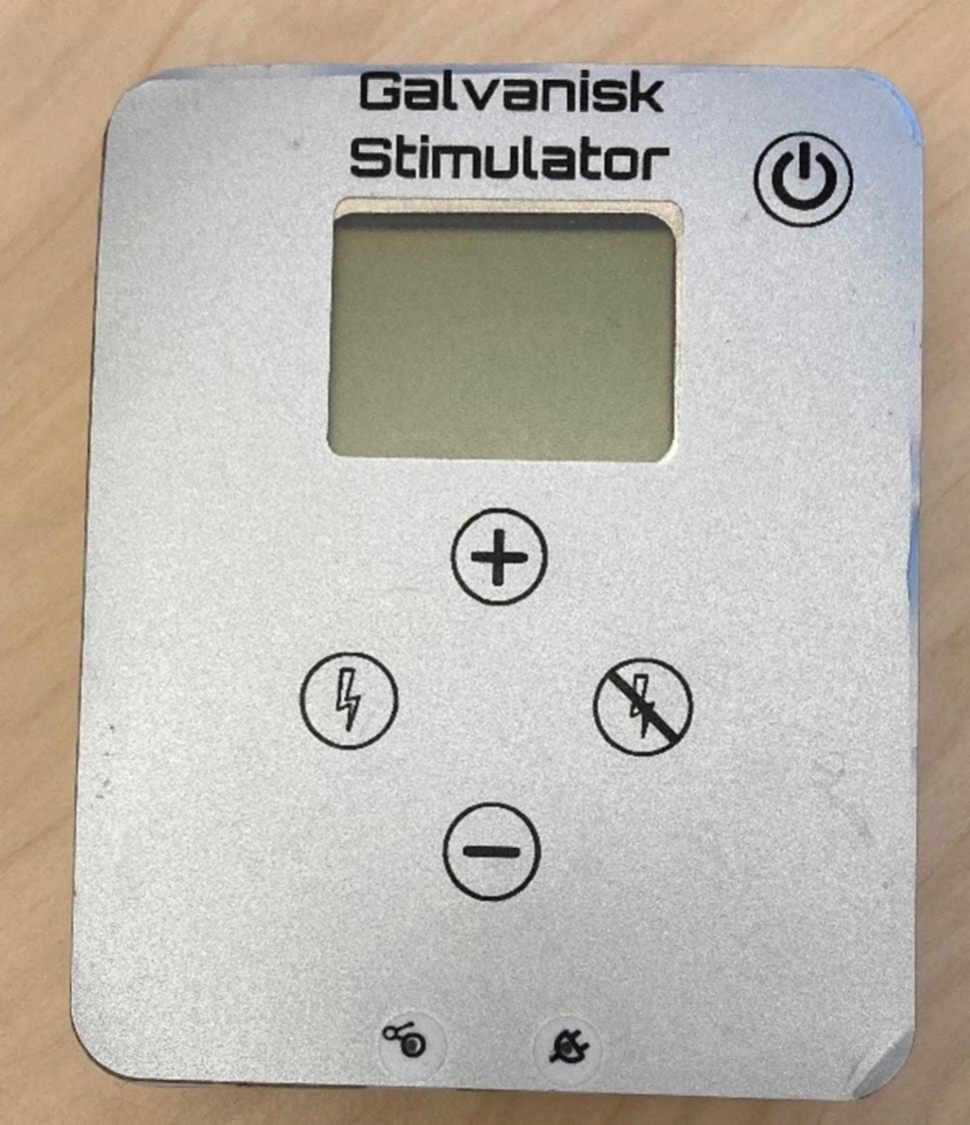
A developed version of Mulavara et al. (2015) Stochastic Vestibular Stimulator, developed by Nigul Ilves, Ilves Engineering

During the tasks, participants had electrodes bilaterally placed on the mastoid bone behind their ears. These electrodes were connected to a portable, programmable stochastic vestibular stimulator, designed to deliver a precisely controlled weak current. The device, see Fig. 2, has previously been used in another study on vestibular stimulation (Samoudi et al. 2015) and is based on the design of a NASA prototype (Mulavara et al. 2015).

The stimulation protocol for the pre-generated stochastic current was similar to the SVS protocol described in Forbes et al. (2014). Like Forbes et al. (2014), the SVS in this study consisted of Gaussian white noise lowpass filtered with a fourth order Butterworth with a cutoff frequency of 25 Hz. However, Forbes et al. (2014) original protocol was modified in two ways to be compatible with our device: (1) time-scaling, and (2) limiting the current amplitude to a range of ±500 µA. The time-scaling was made to retain all attributes (frequency distribution etc.) of the original SVS protocol from Forbes et al. (2014). The decision to limit the amplitude to a 500 µA threshold was based on an in-house pilot study, which aimed to find the maximum undetectable current level. This was crucial in ensuring the double-blinded nature of the procedure. The SVS was delivered at 100 Hz.

For the comfort of the participant, and to further hide the activation of the stimulation, a three second ramp up time was introduced to the current. The same electrical stimulation was applied to all participants and was started at the same time they started the first task.

For further information regarding the protocol the reader is encouraged to read Forbes et al. (2014) study, which covers the nature of the protocol in depth. Information on time and frequency domain of the modified SVS protocol used in this study is available as supplementary information, see S1 and S2.

#### Procedure

All tasks were performed in a quiet room, at either the child psychiatry unit or the participants school, on two different occasions. Only the participant and the experimenter were present in the room when the study was performed at school, while parents were present at the child psychiatry unit. The time between the two occasions varied from 1 to 32 days (median = 7, SD = 7.7). The study was conducted between 2020 and 2022. Participants were assessed individually and placed in a chair, approximately 40 cm from the screen of a 15” Dell XPS 15 9560 laptop with Windows 10.

The skin behind the participants ears were thoroughly cleaned with alcohol (70%) and Cefar Dura-Stick Plus electrodes 5 × 5 cm (www.cefarcompex.com) were applied with a generous amount of Cefar blagel (www.cefarcompex.com) before placed on the skin of the participant. The electrodes were held in place with a custom-made headband worn by the participant.

The occasion of the electrical stimulation (first or second) was chosen randomly and counterbalanced between participants; assessments were double blinded. Thus, neither the participant nor the study leader knew if the protocol applied was sham or real. Participants were asked to report any sensations from the electrodes.

All tasks were developed in PsychoPy standalone (Peirce et al. 2019), version 3.1.0 and were completed in the same order (Spanboard, Word Recall and N-back task), using a Logitech G203 Prodigy mouse. Each test occasion lasted approximately 45 min.

### Data analysis

Analyses were conducted in R (R Core Team 2022), version 2022.02.1, using afex (Singmann et al. 2022), version 1.1-1, and lme4 (Bates et al. 2015), version 1.1-29, to perform linear mixed effects analyses. The analyses were done separately for each task and investigated either the relationship between performance or RT variability, i.e., the standard deviation of reaction time values, and the fixed effects of SVS, group, and the interaction between SVS and group. As random effects, we used intercepts for subjects. There was a significant difference in age and sex between groups, see Table 1 for exact figures. Age and sex were therefore included in all linear mixed effects models as covariates.

All RT values above or equal to 3 SD were removed. The remaining reaction times were then used to compute a RT variability variable for each participant and test, for the different occasions. The data from the three levels of the N-back task (0-, 1- and 2-back) were merged into one “N-back” analysis.

All data were checked for learning effects between the two test occasions. A counter balanced study design ensured that we could isolate the learning effects and make sure that any effects found between SVS on and off were due to the stimulation. The analyses did not reveal any significant differences in either the Spanboard, *t*(138) = − 0.18, *p* = 0.855, Word Recall, *t*(134) = 0.44, *p* = 0.659, or N-back, *t*(137) = 1.56, *p* = 0.122, task.

Visual inspection of residual plots for all analyses revealed deviations from normality and homoscedasticity for the RT variability in the Spanboard task, the performance on the N-back task, and the RT variability in the N-back task. The RT variability in Spanboard and N-back was transformed using square root transformation. Performance on the N-back task was transformed to normality with log-transformation after adding 1 to each subject, since the log(0) is undefined.

Post hoc analyses of pairwise comparisons of the estimated marginal means were done using the emmeans function (Lenth 2022), version 1.7.3, with Bonferroni correction.

## Results

### Spanboard

**Fig. 3:**
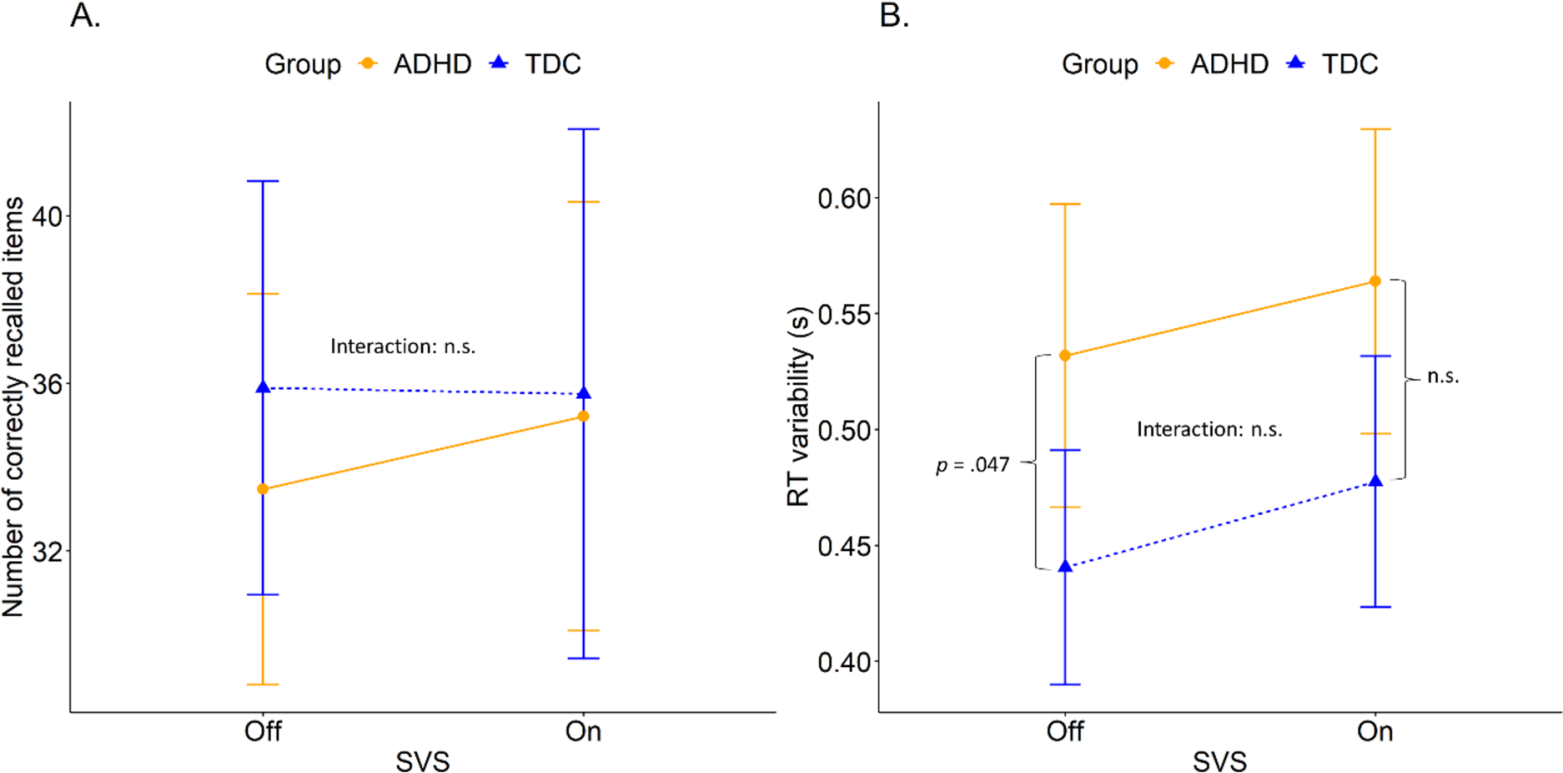
Number of correctly recalled items in a Spanboard task (**A**) and Spanboard RT variability (**B**), as a function of SVS on/off, in two groups: ADHD and TDC. Error bars represent 95% CI. *p*-values are written in the graph for significant differences

*Performance*: There was no interaction between group and SVS and no main effect of either group or SVS (cf. Table 2 and Fig. 3A). There was no difference between SVS *on* (ADHD: M = 35.2, SD = 16.4; TDC: M = 35.8, SD = 16.3) or SVS *off* (ADHD: M = 33.5, SD = 15.0; TDC: M = 35.9, SD = 12.7). However, there was a significant effect of age; the number of correctly recalled dots increased with age.

**Table 2:** Result of linear mixed effect analyses for the spanboard and word recall task

Dependent variable	Predictors	*β*	*95*% CI	*p*
Correct answers on spanboard task	Group [ADHD]	− 5.16	− 13.07–2.75	0.200
	Age	2.02	0.02–4.03	0.050*
	Sex [Boy]	1.21	− 5.54–7.96	0.723
	SVS [on]	− 0.14	− 4.95–4.66	0.953
	SVS [on] * group [ADHD]	1.93	− 4.27–8.13	0.540
RT variability on spanboard task (s)	Group [ADHD]	0.07	0.00–0.13	0.047*
	Age	− 0.01	− 0.02–0.01	0.427
	Sex [Boy]	− 0.01	− 0.06–0.05	0.770
	SVS [on]	0.03	− 0.02–0.08	0.295
	SVS [on] * group [ADHD]	− 0.01	− 0.07–0.06	0.880
Correctly recalled words on word recall task	Group [ADHD]	0.13	− 1.37–1.63	0.866
	Age	0.32	− 0.06–0.70	0.097
	Sex [Boy]	− 0.32	− 1.59–0.95	0.619
	SVS [on]	0.32	− 0.65–1.29	0.513
	SVS [on] * group [ADHD]	–0.74	− 2.00–0.52	0.247

*CI* confidence interval

*p ≤ 0.05

*RT variability*: There was no interaction between SVS and group and no main effect of SVS. However, there was a significant main effect of group in RT variability, where the ADHD group had a larger RT variability than the TDC group and showed a larger RT variability SD (cf. Table 2).

Post hoc analyses of group and SVS revealed a significant difference in RT variability between the groups when SVS was *off*, β = − 0.07 (SE = 0.03), t(104) = − 2.0, *p* = 0.047, but not *on* (see Fig. 3B). The RT variability was significantly higher for the ADHD group with the SVS *off* (ADHD: M = 0.53s, SD = 0.21s; TDC: M = 0.44s, SD = 0.13s). When the SVS was *on* the RT variability increased for both groups (ADHD: M = 0.56s, SD = 0.21s; TDC: M = 0.48s, SD = 0.14s), and the significant difference between groups was no longer present.

#### Word recall

*Performance*: There was no interaction between SVS and group, and no main effect of SVS or group. The participants recalled about equally number of words with SVS *off* (ADHD: M = 9.1, SD = 3.1; TDC: M = 8.6, SD = 2.6) as with SVS *on* (ADHD: M = 8.7, SD = 3.4; TDC: M = 9.0, SD = 2.0). See Table 2 for exact figures.

#### N-back

*Performance*: In the *N-back task* there was no interaction between SVS and group and no main effect of SVS. However, the analysis revealed a significant main effect of group, the ADHD group made significantly more errors than the TDC group, see Table 3.

**Table 3:** Results on performance and RT variability from linear mixed effect analyses for the N-back (0-, 1-, and 2-back summarized) task

Dependent variable	Predictors	*β*/OR^1^	95% CI	*p*
Performance (errors) on N-back task	Group [ADHD]	0.30	0.01–0.58	0.042*
	Age	− 0.03	− 0.09–0.04	0.458
	Sex [Boy]	− 0.16	− 0.39–0.06	0.154
	SVS [on]	0.00	− 0.23–0.24	0.987
	SVS [on] * Group [ADHD]	− 0.07	− 0.38–0.23	0.636
RT variability (s) on N-back task	Group [ADHD]	0.11	0.02–0.19	0.018*
	Age	− 0.02	− 0.04–0.00	0.031*
	Sex [Boy]	− 0.02	− 0.09–0.05	0.667
	SVS [on]	0.00	− 0.06–0.07	0.922
	SVS [on] * Group [ADHD]	− 0.01	− 0.10–0.07	0.771

*RT* reaction time, *CI* confidence interval, *β* estimate, *OR* odds ratio

*p ≤ 0.05

**Fig. 4:**
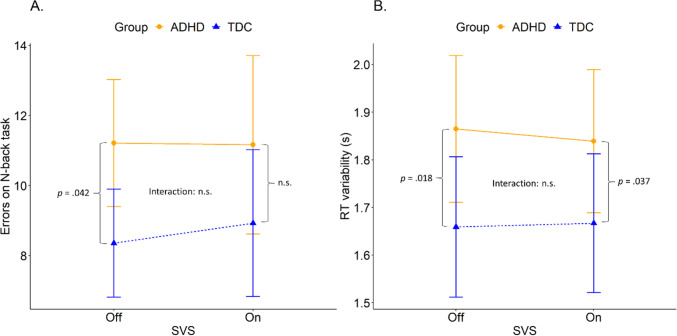
Total number of errors on a N-back (0-, 1-, and 2-back summarized) task (**A**) and N-back RT variability (**B**) as a function of SVS on/off, in two groups: ADHD and TDC. Error bars represent 95% CI. *p*-values are written in the graph for significant differences

Post hoc analyses revealed a significant difference between groups, β = − 0.30 (SE = 0.14), t(112) = − 2.06, *p* = 0.042, with SVS *off*. When SVS was *on* the difference between groups disappeared. This effect is most likely due to both groups having a larger SD with the SVS *on* (ADHD: M = 11.2, SD = 8.2; TDC: M = 8.9, SD = 5.4) compared to *off* (ADHD: M = 11.2, SD = 5.8; TDC: M=8.4, SD = 4.0), see Fig. 4A.

*RT variability*: The analysis of the *N-back task* found no interaction between SVS and group and no main effects of SVS. However, group, β = 0.11 (SE = 0.04), t(106) = 2.40, *p* = 0.018, and age, β = − 0.02 (SE = 0.01), t(67) = − 2.20, *p* = 0.031, were significant. The ADHD group had a larger RT variability than the TDC group, see Fig. 4B.

Post hoc comparisons of SVS (on/off) and group (ADHD/TDC) revealed a significant difference between groups with SVS *off*, β = − 0.11 (SE = 0.04), t(106) = − 2.40, *p* = 0.018. With the SVS *on* there were still a significant difference between groups, β = -0.09, (SE = 0.04), t(106) = − 2.11, *p* = 0.037. That is, the groups differed significantly in their RT variability during both test occasions, and when SVS was *on* the ADHD group had a significantly larger RT variability than the TDC group (ADHD: M = 1.84s, SD = 0.48s; TDC: M = 1.67s, SD = 0.38s), which was also the case when SVS was *off* (ADHD: M = 1.87s, SD = 0.50s; TDC: M = 1.66s, SD = 0.38s). All performance scores can be viewed in S3.

## Discussion

This study explored the effects of stochastic vestibular stimulation (SVS) on working memory in children diagnosed with ADHD and typically developing children (TDC) in three working memory tasks, relying on the MBA model (Sikström and Söderlund 2007) and previous work with auditory noise stimulation (Söderlund et al. 2016). The MBA model is based on the assumption that a certain level of neural activity in the brain is a prerequisite for maximal performance in cognitive tasks and suggest that external sensory noise stimulation should be beneficial for people with attention deficits to compensate for low levels of dopamine (DA) and norepinephrine (NA). In contrast, external sensory noise stimulation would be impairing for TDC; since they already have sufficient levels of DA and NA in the brain, adding additional noise would push them away from the ‘optimal’ operational state since the relation between sensory noise levels and performance follows an inverted U-function and TDC will end up at the far side of the U. In contrast to our predictions (see Fig. 1 for hypotheses), SVS did not improve cognitive performance or decrease reaction time (RT) variability in children with ADHD (H1), nor did it impair the performance for TDC (H2). However, we found partial support for H3, that the TDC group would have higher performance and lower RT variability without SVS compared to the ADHD group (H3a), and that SVS would remove such differences (H3b). When there was a difference between the groups with SVS off (Table 4, H3a : bold numbers), this difference disappeared in two cases (Table 4, H3b : bold numbers) and was primarily due to a higher variability in both groups (cf. Figs. 3B and 4A). Below the implications of these findings are discussed.

**Table 4:** Summary of results in relation to hypotheses

Task	Outcome measure	H1	H2	H3a	H3b
Spanboard	Performance RT variability	*n.s.* *n.s.*	*n.s.* *n.s.*	*n.s.* *p* < .047*	*n.s.* **n.s.**
Word Recall	Performance	*n.s.*	*n.s.*	*n.s.*	*n.s.*
N-back	Performance RT variability	*n.s.* *n.s.*	*n.s.* *n.s.*	*p* < .042* *p* < .018*	**n.s.** p < .037*

Italic values go against our hypotheses, bold values are in line with our hypotheses. H3b is dependent on H3a and can only be fulfilled if H3a is fulfilled

*p ≤ 0.05

Clearly, our data did not support the MBA model’s prediction that SVS would be beneficial for children with ADHD. There are several possible reasons for this. Although previous work has shown that auditory noise stimulation improves learning, executive functioning and episodic- and working memory (Angwin et al. 2019; Helps et al. 2014; Söderlund et al. 2016), studies with vestibular stimulation show more mixed results. A review on vestibular stimulation shows that effects on cognition are both varying and inconsistent (Bigelow and Agrawal 2015). Consequently, it is unclear whether the MBA model is applicable to all noise modalities and whether the effects found for auditory noise stimulation generalize to SVS. We found no support for this in the present study, and it is questionable whether SVS can be introduced into the neural system to affect higher cognition, such as performance.

Studying the effect of SVS on cognition in children with ADHD is methodologically challenging for a number of reasons, and there are many open questions that should be addressed in future research. Here we discuss three such questions. First, what is the optimal stimulation protocol? In the present study we used the same level of noise for all individuals, but not only individual differences may influence the degree to which the noise reaches the vestibular system, several other parameters also have the potential to influence the efficacy of the stimulation (McLaren et al. 2023). Other studies using SVS have, for example, used thresholds to evaluate the individual optimal level of noise for each subject (Samoudi et al. 2015).

Second, a previous review suggests that some tasks are more responsive to noise than other (Pickens et al. 2019). The present study used two tasks (Spanboard, Word Recall) that have previously shown significant effects on performance in auditory noise stimulation in children with ADHD (Helps et al. 2014; Söderlund et al. 2016) and visuospatial tasks, such as the Spanboard task, is suggested to be one of the most sensitive measures of working memory deficits in ADHD (Martinussen et al. 2005; Westerberg et al. 2004). However, in contrast to our hypothesis, the two groups in our study did not display any significant difference in performance in any of the tasks.

This leads to the third question, who are noise benefiters? ADHD is a heterogeneous group with comorbidities and neurodiversity, and resent research emphasizes the necessity to take this heterogeneity into account (Cortese et al. 2022; Sonuga-Barke and Thapar 2021). As such, there may be subgroups under the large ADHD-umbrella that are differentially responsive to noise, and therefore perform better or worse due to noise exposure.

Here we identify a limitation of the present study, not screening for vestibular dysfunction, since there is an increased prevalence in ADHD (Caldani et al. 2020; Feng et al. 2007; Franco and Panhoca 2008) and vestibular rehabilitation programs have shown improvements in cognitive performance in this group (Lotfi et al. 2017). This is a subgroup of ADHD that might experience different effects of the SVS than one without vestibular dysfunction. Also, the wide age range in the ADHD sample (8–17 years old) might reduce the statistical power to detect between-group differences, therefore we included age in all linear regression analyses. Another limitation is that, unfortunately, the auditory noise stimulation in one of our conditions failed and therefore prevented us from drawing any conclusions about the potential effects of auditory noise stimulation and its interaction with SVS.

Finally, we urge future research to explore: (i) other cognitive tasks, (ii) the use of several stimulation protocols including higher current levels, and (iii) different target groups to be able to make firm conclusions regarding vestibular stimulation and cognitive performance for different groups of people.

## Conclusions

Stochastic vestibular stimulation (SVS) has been applied to children with ADHD to investigate whether it improves cognitive performance and decreases reaction time (RT) variability. In contrast to the predictions of the MBA model, SVS did not affect cognitive performance or RT variability in children with ADHD or a group of typically developing children (TDC). Consequently, the previously reported beneficial effect of auditory noise exposure in children with ADHD may not generalize to vestibular noise stimulation. This highlights that SVS may not be suitable as a complementary treatment for children with ADHD. However, data showed marginal effects on RT variability and performance in two tests, in accordance with predictions, that provide arguments for further exploration of vestibular stimulation.

### Supplementary Information

Below is the link to the electronic supplementary material.

Supplementary file1 (PNG 41 KB)

Supplementary file2 (PNG 56 KB)

Supplementary file3 (DOCX 15 KB)

## Data Availability

The data from the current study are available from the corresponding author on request.
