# Supplementary figures and images for: Effects of stochastic vestibular stimulation on cognitive performance in children with ADHD
Source: Exp Brain Res. 2023 Oct 9;241(11-12):2693–703. doi: 10.1007/s00221-023-06713-7 (PMC10635964; doi:10.1007/s00221-023-06713-7)

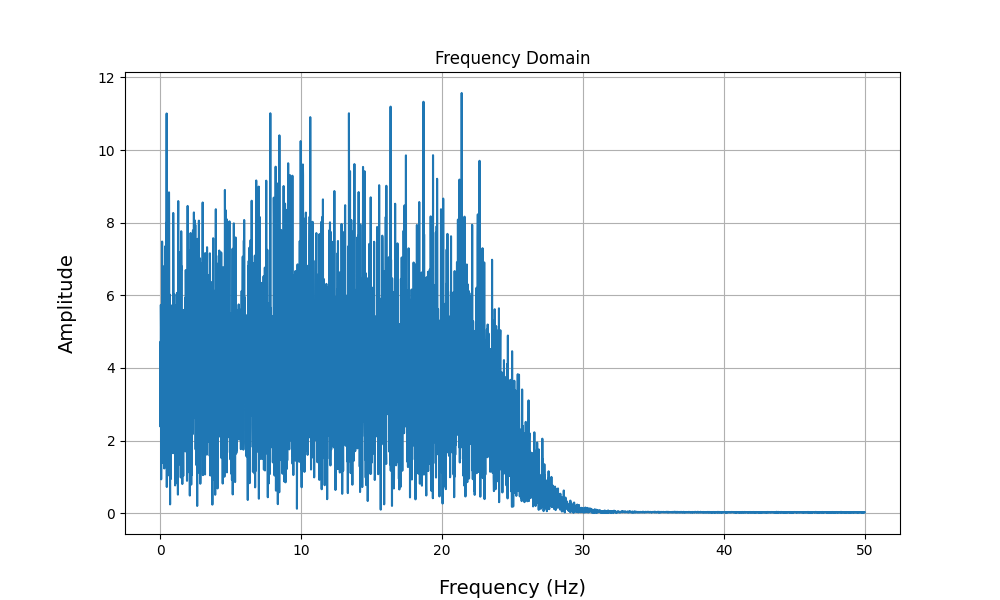

Supplement: Supplementary file 1 — Supplementary file1 (PNG 41 KB) [file 221_2023_6713_MOESM1_ESM.png]

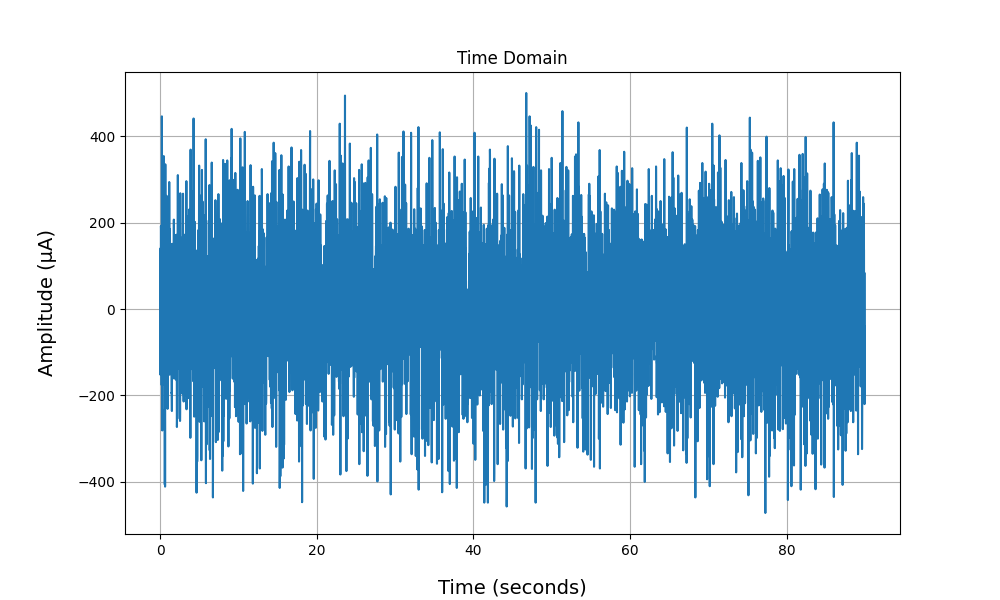

Supplement: Supplementary file 2 — Supplementary file2 (PNG 56 KB) [file 221_2023_6713_MOESM2_ESM.png]
