# Supplementary material for: Effects of stochastic vestibular stimulation on cognitive performance in children with ADHD
Source: Exp Brain Res. 2023 Oct 9;241(11-12):2693–703. doi: 10.1007/s00221-023-06713-7 (PMC10635964; doi:10.1007/s00221-023-06713-7)
Supplement: Supplementary file 3 — Supplementary file3 (DOCX 15 KB) [file 221_2023_6713_MOESM3_ESM.docx]

| Table S3. Participants’ performance and RT variability on the Spanboard, Word Recall and N-back taks. | | | | | |
| --- | --- | --- | --- | --- | --- |
|  |  | SVS off | | SVS on | |
|  |  | ADHD | TDC | ADHD | TDC |
| Spanboard | Performance (correct answers) | 33.5 (15.0) | 35.9 (12.7) | 35.2 (16.4) | 35.8 (16.3) |
|  | RT variability (s) | 0.53 (0.21) | 0.44 (0.13) | 0.56 (0.21) | 0.48 (0.14) |
| Word Recall | Performance (correct answers) | 9.1 (3.1) | 8.6 (2.6) | 8.7 (3.4) | 9.0 (2.0) |
| N-back | Performance (errors) | 11.2 (5.8) | 8.4 (4.0) | 11.2 (8.2) | 8.9 (5.4) |
|  | RT variability (s) | 1.87 (0.50) | 1.66 (0.38) | 1.84 (0.48) | 1.67 (0.38) |

Mean (standard deviation).
